# A bibliometric analysis of the Mediterranean diet in metabolic syndrome (2015–2025)

**DOI:** 10.3389/fnut.2026.1765074

**Published:** 2026-01-16

**Authors:** Xuefeng Xi, Shiliang Hu, Wenlong Hou, Yuanyuan Qin, Chu Wu, Li Luo, Shuai Hu

**Affiliations:** 1 College of Martial Arts, Henan University, Kaifeng, China; 2 Physical Education and Sports School of Soochow University, Suzhou, China; 3 Suzhou TCM Hospital Affiliated to Nanjing University of Chinese Medicine, Suzhou, China

**Keywords:** bibliometric analysis, cardiovascular diseases, Mediterranean diet, metabolic syndrome, non-alcoholic fatty liver disease, type 2 diabetes mellitus

## Abstract

**Objective:**

The Mediterranean diet (MD), a widely recognized healthy dietary pattern, has demonstrated significant value in the prevention and management of metabolic syndrome (MetS). However, quantitative integrated analyses of its mechanisms of action remain scarce, highlighting an urgent need for systematic collation.

**Methods:**

This study retrieved relevant literature published between 2015 and 2025 from the Web of Science and Scopus databases, and conducted visual bibliometric analyses using R software (Bibliometrix package), VOSviewer, and CiteSpace.

**Results:**

A total of 1723 valid articles were identified from the Web of Science, and 1061 from Scopus. The number of publications steadily increased from 2015 to 2022, with a particularly notable growth spurt between 2016 and 2018, followed by a slight decline from 2022 to 2024. Spain led significantly in the number of publications, followed by Italy, the United States, and Iran, and Spain has established an extensive international cooperation network. At the journal level, Nutrients serves as the core academic platform in this field, ranking first in both publication volume and citation frequency. Current research hotspots primarily focus on the regulatory effects of MD on blood glucose homeostasis, insulin sensitivity, lipid metabolism, and blood pressure in MetS patients, as well as the mechanisms by which bioactive components (e.g., polyphenols, unsaturated fatty acids, and vitamins) promote metabolic health through anti-inflammatory, antioxidant, and insulin-sensitizing pathways. Gut microbiota modulation has evolved into an emerging research direction in this domain.

**Conclusion:**

Over the past decade, interdisciplinary research on MD and MetS has gained increasing attention and is expected to become a core focus in the non-pharmacological intervention of MetS. This study systematically clarifies the research status, hot topics, and developmental context of this field, providing important references for future precision nutrition mechanism research and clinical intervention trials.

## 1 Introduction

Metabolic syndrome (MetS) is a prevalent cluster of metabolic disorders that poses a serious threat to human health. Its core features include interconnected risk factors such as abdominal obesity, insulin resistance, hyperglycemia, dyslipidemia, and hypertension (1). These metabolic abnormalities significantly increase the risk of various chronic diseases, including cardiovascular diseases, type 2 diabetes mellitus (T2DM), non-alcoholic fatty liver disease (NAFLD), and cognitive impairment (2). Although there is ongoing debate regarding a unified definition of MetS, indicators such as blood glucose levels, central obesity, blood pressure, triglycerides, and high-density lipoprotein cholesterol are widely used for clinical assessment and diagnosis (3). In recent years, the global prevalence of MetS has continued to rise, becoming a major public health concern. Currently, approximately 25% of the global population meets the diagnostic criteria for MetS (4). Data from the National Health and Nutrition Examination Survey (NHANES 2011—2018) show that the prevalence of MetS among American adults is as high as 39.8% (5), while in China, this proportion is approximately 24.2% (6). Both developed and developing countries are severely affected, mainly due to unhealthy lifestyles, population aging, and rising obesity rates (7). MetS not only severely impairs the quality of life of affected individuals but also imposes a heavy socioeconomic burden. Current prevention and treatment strategies mainly rely on pharmacotherapy and lifestyle interventions, but their efficacy varies greatly among individuals, and drug-related adverse reactions limit long-term application (8). Therefore, there is an urgent need to explore safe, economical, and sustainable novel intervention strategies to improve MetS-related metabolic abnormalities and reduce the risk of complications.

The Mediterranean diet (MD) is a representative dietary pattern originating from countries bordering the Mediterranean Sea. It is dominated by plant-based foods such as vegetables, fruits, whole grains, legumes, and nuts; uses olive oil as the main fat source; includes moderate intake of fish, poultry, and dairy products; and limits the consumption of red meat and processed foods (9). This dietary pattern provides abundant dietary fiber, monounsaturated fatty acids, vitamins, and minerals, ensuring comprehensive nutrient supply. In recent years, the MD has attracted considerable attention due to its potential benefits in weight management and the prevention and control of metabolic diseases. Studies have shown that the MD can regulate the expression of specific microRNAs (e.g., miR182), thereby playing a key role in maintaining blood glucose homeostasis and improving central obesity (10). Clinical data on diabetic patients indicate that the MD can significantly reduce circulating C-reactive protein (CRP) levels, upregulate adiponectin expression, improve systemic inflammatory status, enhance insulin sensitivity, and optimize the Homeostatic Model Assessment of Insulin Resistance (HOMA) index (11). For patients with cardiovascular diseases, adhering to the MD can effectively lower blood pressure and low-density lipoprotein cholesterol (LDL-C) levels, reduce visceral fat accumulation, and decrease body weight (12). The health benefits of the MD cover multiple areas: it helps improve liver lipid metabolism and reduce the risk of NAFLD (13); its antioxidant and anti-inflammatory components can alleviate age-related brain atrophy and exert neuroprotective effects (14); it can reduce the frequency and intensity of attacks in patients with chronic migraine (15); and it may also show potential value in cancer prevention, recurrence control, and prolonging patient survival by inhibiting oxidative stress, inflammatory responses, and DNA damage (16). Despite the various health benefits of the MD, its long-term safety and feasibility in the management of MetS remain controversial. Excessive intake of high-energy foods may lead to calorie surplus, increasing the risk of overweight and dyslipidemia, which is particularly prominent in individuals with insufficient physical activity or low metabolic rates (17). In addition, red wine, a component of the MD, may further exacerbate liver damage and metabolic disorders in patients with impaired liver function (18). Notably, there is a lack of systematic analysis of research hotspots and cutting-edge directions in this field, highlighting the need to conduct scientometric and visualization studies to clarify future research priorities and developmental directions.

As a systematic quantitative research method, bibliometric analysis can be used to evaluate academic literature related to a specific field (19–21). Compared with traditional systematic literature review methods, bibliometric analysis has distinct advantages. Specifically, by collecting and analyzing data such as the number of publications, citation frequency, journals, research institutions, and keywords, this method identifies the core developmental patterns, influential research forces, and dynamically evolving research topics in the field, as well as explores emerging research directions and existing literature gaps. Although a large number of literatures on the MD in the field of MetS have been published in recent years, no researchers have conducted a bibliometric analysis on this field. Therefore, the research hotspots and trends in this field remain unclear. This study conducted a bibliometric analysis of literature related to the MD and MetS published between 2015 and 2025, aiming to comprehensively present the research trends, hot topics, and frontiers in this field, and to provide a reference for dietary interventions for MetS and the development of novel therapeutic strategies.

## 2 Materials and methods

### 2.1 Literature sources and search strategy

A comprehensive literature retrieval was performed on October 21, 2022, in two major academic databases: the Web of Science Core Collection (WoSCC) and Scopus. For the WoSCC database, the search query was defined as follows: TS = (“Mediterranean diet” OR “Mediterranean dietary pattern”) AND TS = (“Metabolic Syndrome” OR “Syndrome, Metabolic” OR “Insulin Resistance Syndrome” OR “Dysmetabolic Syndrome” OR “Reaven Syndrome” OR “Metabolic Cardiovascular Syndrome”) AND PY = (2015–2025) AND DT = (Article OR Review) AND LA = (English). For the Scopus database, the retrieval strategy was formulated as: TITLE-ABS-KEY(“Mediterranean diet” OR “Mediterranean dietary pattern”) AND TITLE-ABS-KEY(“Metabolic Syndrome” OR “Syndrome, Metabolic” OR “Insulin Resistance Syndrome” OR “Dysmetabolic Syndrome” OR “Reaven Syndrome” OR “Metabolic Cardiovascular Syndrome”) AND PUBYEAR > 2014 AND PUBYEAR < 2026 AND (LIMIT-TO(DOCTYPE, “ar”) OR LIMIT-TO(DOCTYPE, “re”)) AND (LIMIT-TO(LANGUAGE, “English”)). Following the removal of duplicate entries and irrelevant documents, 1723 and 1,061 valid literatures were ultimately included from WoSCC and Scopus, respectively. Complete records (including citation information) from WoSCC were exported in plain-text format, while full records with citations from Scopus were exported in CSV format to ensure data integrity. Since the core analysis of this study primarily relies on data from the WoSCC database, the detailed retrieval strategy and data filtering process for this database are elaborated below.

### 2.2 Data analysis

Due to the inherent differences in data structures between WoSCC and Scopus, direct merging would lead to the loss of key information. Therefore, the data from the two databases were analyzed independently to ensure the reliability of the results. Notably, considering the academic quality and authority of the literature indexed in WoSCC, subsequent core analyses mainly focused on the data from this database. The analyses of Scopus data (including annual publication trends and keyword clustering) are presented in Supplementary material for reference. Data analysis tools and their specific applications were as follows: The Origin 2018 software was used for the statistical analysis of annual publication volumes. The R language Bibliometrix package^1^ (version 4.3.1), VOSviewer software (version 1.6.18), and CiteSpace software (version 6.3.1.0) were employed for data visualization and scientific knowledge mapping.

**Figure 1:**
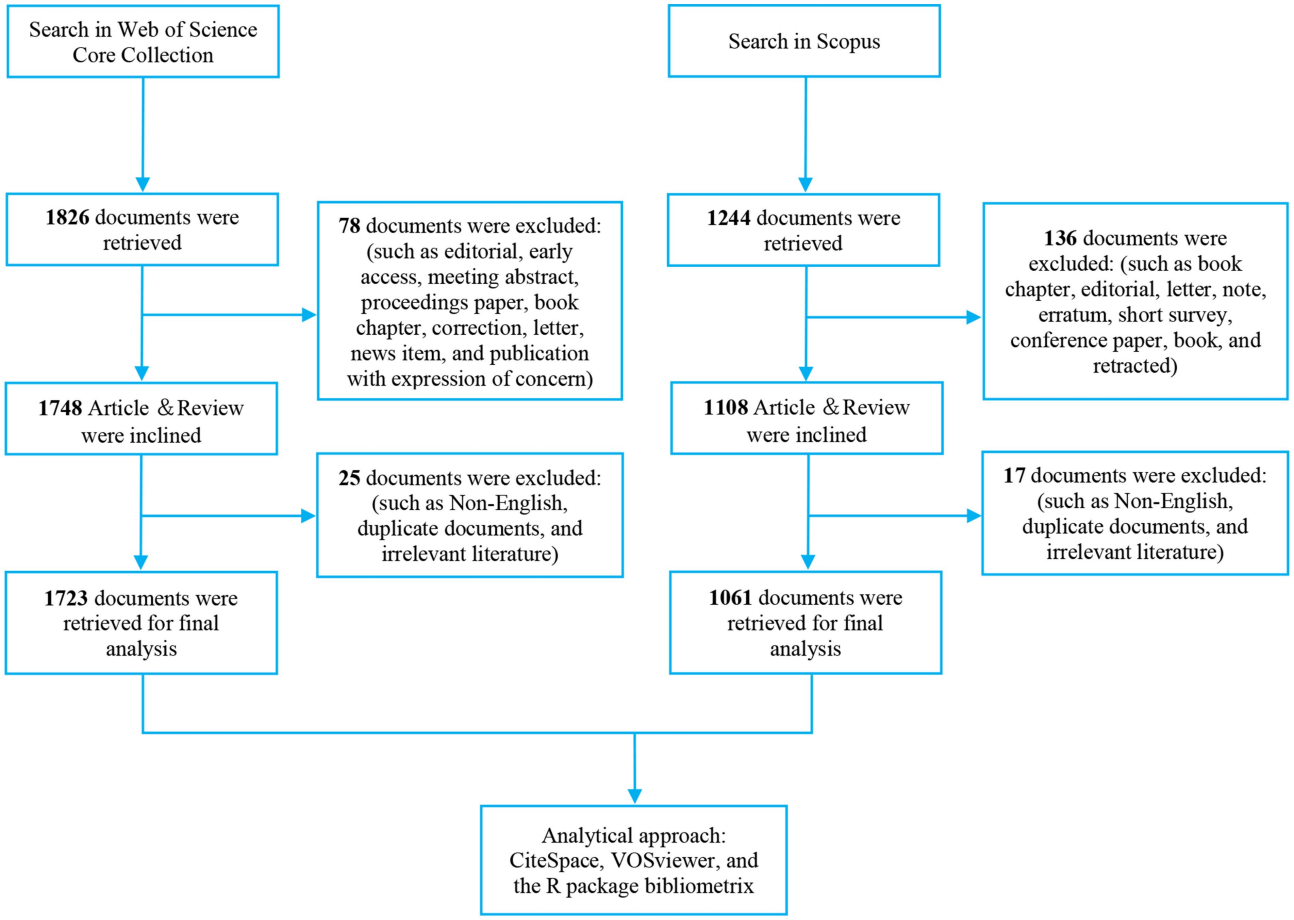
Flowchart of the publications selection.

VOSviewer was used to construct national collaboration networks, conduct source co-citation analysis, and perform keyword co-occurrence analysis with the following parameters: the minimum number of documents per country≥14; the minimum number of documents per institution≥10; the minimum citation count≥152 for co-citation analysis; and the keyword occurrence frequency≥15 (excluding “Mediterranean diet,” “metabolic syndrome,” and their synonyms). CiteSpace software was used to identify the top 25 documents with the strongest citation bursts. CiteSpace parameters were set as follows: time slicing (2015–2025), years per slice (1), node type (cited reference), selection criteria (top N = 50), and no clipping. The Journal Impact Factor (IF) data were obtained from the 2024 Journal Citation Reports (JCR). Figure 1 shows the flowchart for search strategy and selection process in this study.

## 3 Results

### 3.1 Overview of selected studies on MD in MetS

**Figure 2:**
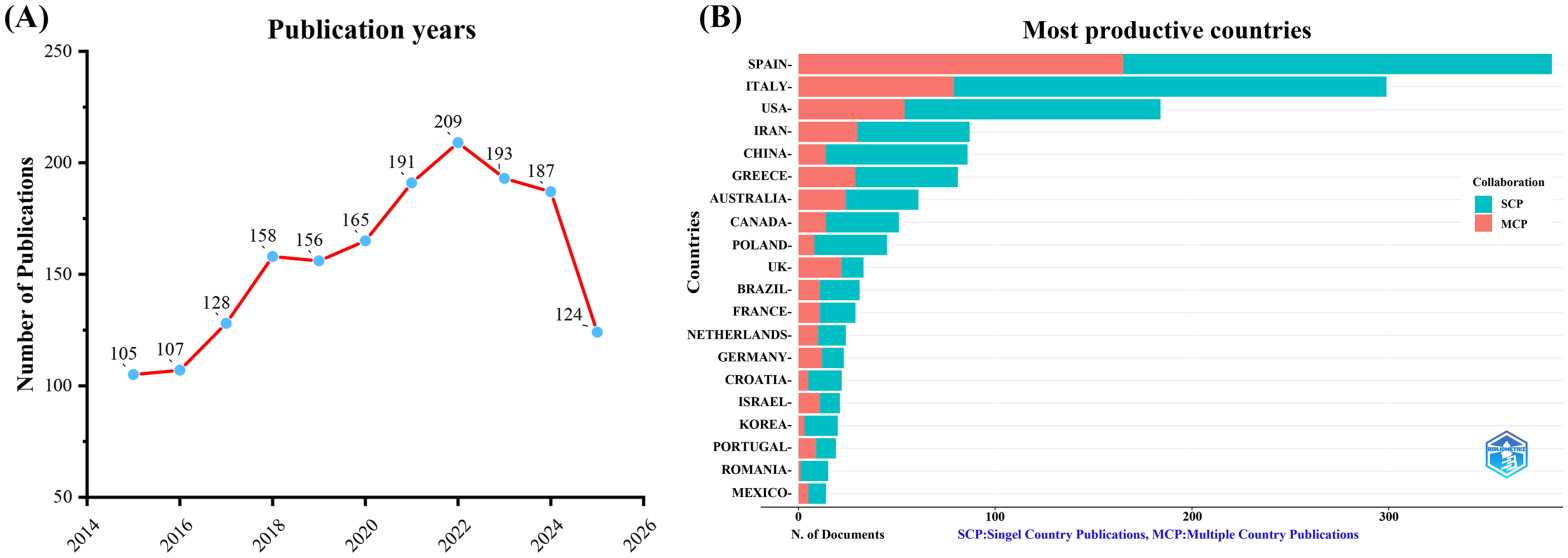
Trends in annual publication outputs on MD in metabolic syndrome from 2015 to 2025. **(A)** Trends of annual publication outputs and **(B)** Distribution of corresponding authors’ countries and cooperation.

A total of 1723 unique documents were retrieved from WoSCC. As shown in Figure 2A, the annual publication volume in this field showed an overall upward trend from 2015 to 2025, with a particularly rapid growth period from 2016 to 2018. The publication volume reached its peak in 2022, with 209 articles. This growth trend reflects the vitality and in—depth nature of research in the field. However, from 2022 to 2024, it entered an adjustment and fluctuation phase, with a slight decline in publication volume, which still remained above 180 articles. The reason for the decrease in the number of publications may be attributed to specific years and database delays. As of October 21, 2025, 124 papers had been published in the field, further contributing to the accumulation of relevant literature. After deduplication, 1,061 unique records were collected from Scopus. The trend in publication volume was consistent with that of WoSCC (Supplementary Figure S1), indicating a growing research interest in the association between MD and MetS.

**Figure 3:**
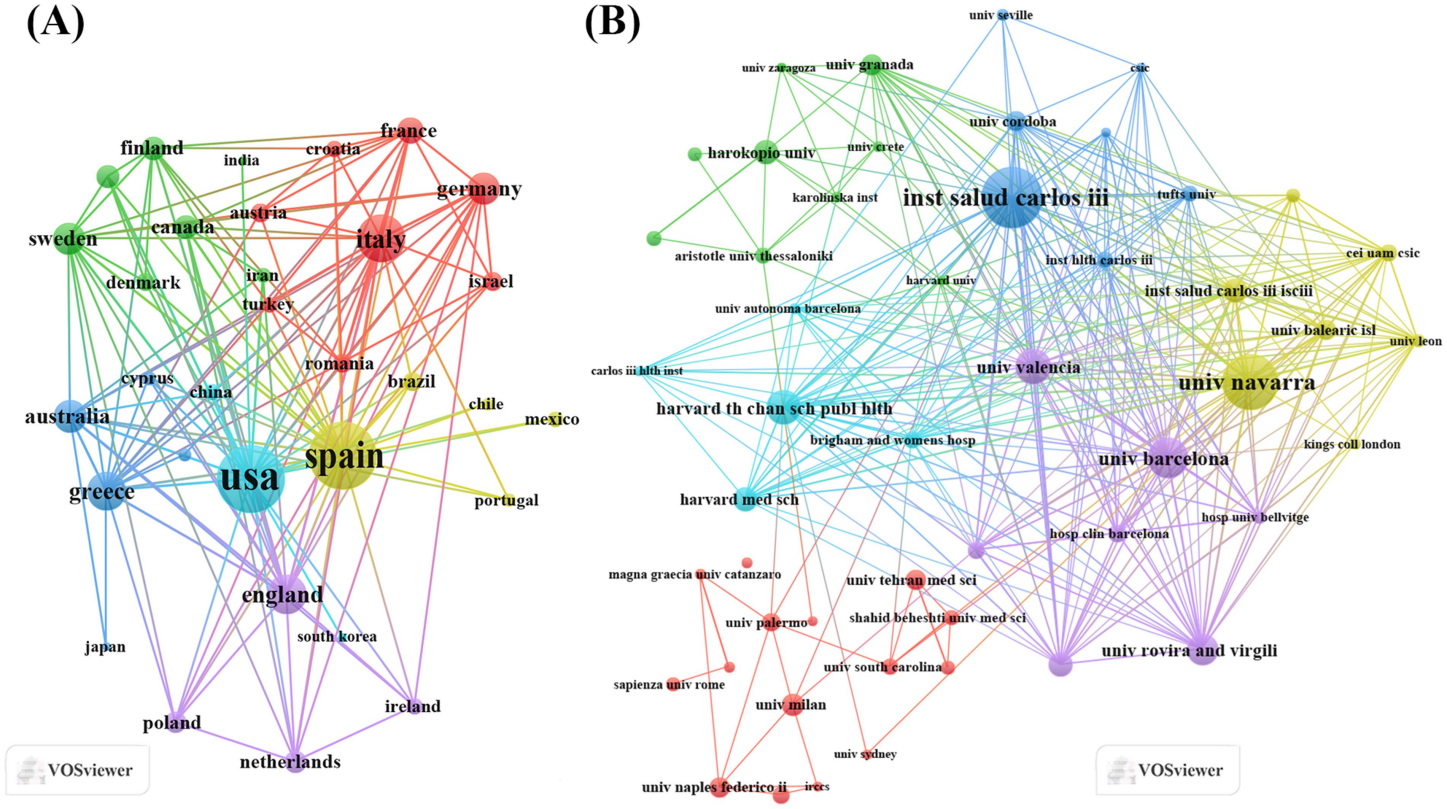
Map of countries/regions and institutions involved in MD in metabolic syndrome research from 2015 to 2025. **(A)** Map of international cooperation networks and **(B)** map of institutional cooperation networks.

Analysis of the countries of corresponding authors revealed that Spain (*n* = 383) had the highest number of publications, followed by Italy (*n* = 299), the United States (*n* = 184), Iran (*n* = 87), and China (*n* = 86). Spain’s leading publication output underscores its prominent position and significant contributions in the field. Additionally, among the top 10 countries, Spain (43.1%) and Australia (39.30%) had the highest proportions of multi-country publications (MCPs), indicating a strong emphasis on international cooperation and academic exchange in these two countries (Figure 2B; Table 1). Figure 3A shows that the United States serves as the collaboration hub in the field, highlighting its crucial role in advancing research. Among the top 10 publishing institutions, 7 were from Spain, with 1 each from the United States, Greece, and Italy. Spain’s Carlos III Health Institute ranked first with 175 papers, followed by the University of Navarra (Spain, 121 papers) and Harvard University (United States, 109 papers). These data indicate that research institutions in Spain place greater emphasis on investigating the effects of MD on MetS, potentially due to Spain’s status as a Mediterranean coastal country. Its dietary habits, which primarily include fruits, vegetables, fish, whole grains, legumes, and olive oil, align closely with the core components of MD (Figure 3B; Table 2).

**Table 1:** Most relevant countries by corresponding authors of MD in metabolic syndrome.

Country	Articles	SCP	MCP	Freq (%)	MCP_ratio (%)
Spain	383	218	165	22.2	43.1
Italy	299	220	79	17.4	26.4
USA	184	130	54	10.7	29.3
Iran	87	57	30	5	34.5
China	86	72	14	5	16.3
Greece	81	52	29	4.7	35.8
Australia	61	37	24	3.5	39.3
Canada	51	37	14	3	27.5
Poland	45	2.6	37	8	17.8
United Kingdom	33	1.9	11	22	66.7
Brazil	31	1.8	20	11	35.5
France	29	1.7	18	11	37.9
Netherlands	24	1.4	14	10	41.7
Germany	23	1.3	11	12	52.2
Croatia	22	1.3	17	5	22.7
Israel	21	1.2	10	11	52.4
Korea	20	1.2	17	3	15
Portugal	19	1.1	10	9	47.4
Romania	15	0.9	14	1	6.7
Chile	12	0.7	8	4	33.3

MCP, Multiple country publication; SCP, Single country publication.

**Table 2:** Top 10 most relevant affiliations of MD in metabolic syndrome.

Rank	Affiliation	Articles (*n*)
1	Carlos III Health Institute	175
2	University of Navarra	121
3	Harvard University	109
4	University of Barcelona	82
5	University of Valencia	68
6	Rovira i Virgili University	60
7	Harokopio University	44
8	University of Malaga	42
9	University of Milan	39
10	University of Granada	37

### 3.2 Journal analysis and visualization

The R language Bibliometrix package was utilized to analyze journals with the highest publication volume and citation contributions in the MD and MetS field, and graphs were plotted using the ggplot2 package. VOSviewer was employed for journal co - citation analysis.

**Figure 4:**
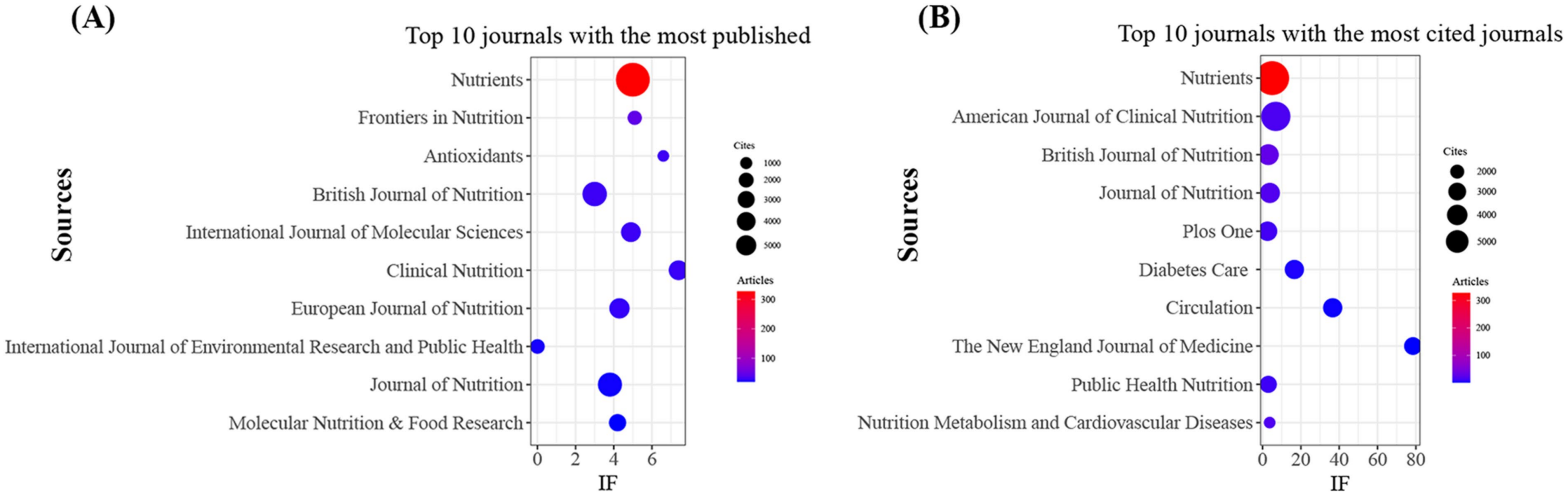
Journals with the highest publication volume and citation counts. **(A)** Journals with the highest publication volume and **(B)** Journals with the highest citation counts.

**Figure 5:**
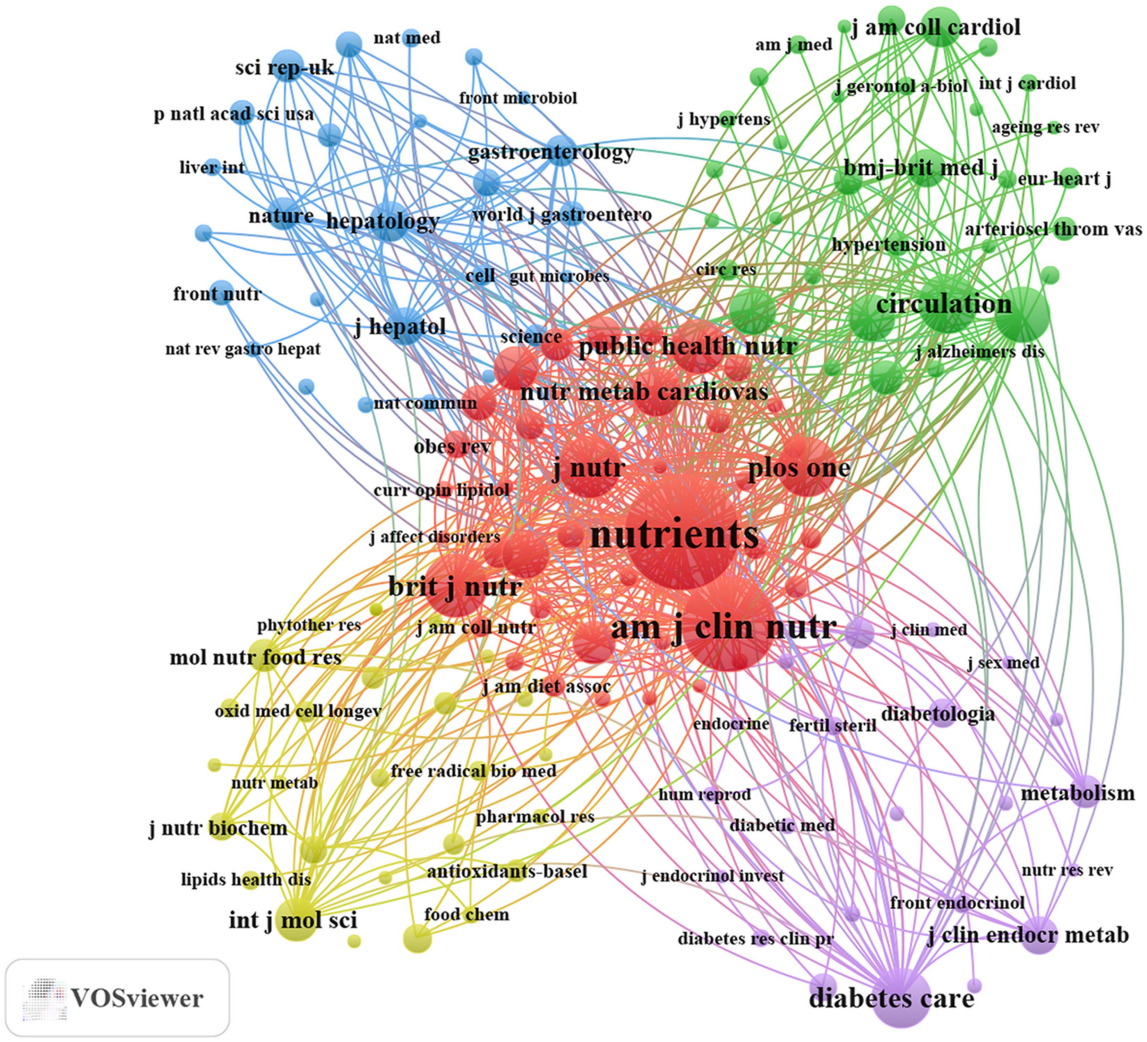
Co-cited journals related to MD in metabolic syndrome from 2015 to 2025.

The 1723 documents included in this study were published in 567 academic journals (Supplementary material 1). As shown in Table 2 and Figure 4A, *Nutrients* had the highest publication volume (*n* = 327, IF = 5), followed by *Frontiers in Nutrition* (*n* = 47, IF = 5.1), *Antioxidants* (*n* = 31, IF = 6.6), *British Journal of Nutrition* (*n* = 30, IF = 3), and *International Journal of Molecular Sciences* (*n* = 30, IF = 4.9). Table 3 and Figure 4B present the journals with the highest citation counts, including *Nutrients* (*n* = 5,807, IF = 5), *American Journal of Clinical Nutrition* (*n* = 4,187, IF = 6.9), *British Journal of Nutrition* (*n* = 2,185, IF = 3), *The Journal of Nutrition* (*n* = 2081, IF = 3.8), and *PLoS One* (*n* = 1970, IF = 2.6). Notably, the journal co-citation map in Figure 5 shows that *Nutrients*, *American Journal of Clinical Nutrition*, and *British Journal of Nutrition* are core collaboration hubs. These findings collectively demonstrate the significant influence of *Nutrients* in the MD and MetS research field (see Table 4).

**Table 3:** Top 10 journals with the most published articles.

Rank	Journal	Articles	Cites	IF (2024)
1	Nutrients	327	5,807	5
2	Frontiers in Nutrition	47	451	5.1
3	Antioxidants	31	372	6.6
4	British Journal of Nutrition	30	2,185	3
5	International Journal of Molecular Sciences	30	1,118	4.9
6	Clinical Nutrition	29	1,102	7.4
7	European Journal of Nutrition	28	1,209	4.3
8	International journal of environmental research and public health	22	474	0
9	Journal of nutrition	21	2081	3.8
10	Molecular nutrition and food research	20	720	4.2

**Table 4:** Top 10 journals with the most cited journals.

Rank	Journal	Cites	Articles	IF (2024)
1	Nutrients	5,807	327	5
2	American Journal of Clinical Nutrition	4,187	18	6.9
3	British Journal of Nutrition	2,185	30	3
4	Journal of Nutrition	2081	21	3.8
5	Plos One	1970	14	2.6
6	Diabetes Care	1944	4	16.6
7	Circulation	1940	2	36.6
8	The New England Journal of Medicine	1763	1	78.5
9	Public Health Nutrition	1,696	12	3
10	Nutrition Metabolism and Cardiovascular Diseases	1,437	20	3.7

### 3.3 Most cited references and reference burst

Using the R language Bibliometrix package, we identified the top 25 most cited documents in the MD and MetS field (Table 5). The titles of these citations, along with their respective DOI, are listed in Supplementary material 2. All documents had more than 260 citations and were distributed across 23 different journals, reflecting the lack of a highly centralized research system and the potential for further exploration in the field. Notably, although Nature Reviews Endocrinology had only one document included in the top-cited list, it contributed the highest single - document citation count, indirectly confirming its authoritative influence in the field. Overall, no single journal dominates the field, and the distribution of journals is relatively scattered. The top three most cited documents were: “Global aetiology and epidemiology of type 2 diabetes mellitus and its complications,” “Primary Prevention of Cardiovascular Disease with a Mediterranean Diet Supplemented with Extra-Virgin Olive Oil or Nuts,” and “Mediterranean diet and multiple health outcomes: an umbrella review of meta - analyses of observational studies and randomized trials.” Further analysis indicated that these highly cited documents primarily focus on macro reviews of the field, lacking in - depth analysis of specific research directions.

**Table 5:** Top 25 cited references related to MD in metabolic syndrome.

Paper	DOI	Total citations	TC per year
Zheng Y, 2018, Nat Rev. Endocrinol	10.1038/nrendo.2017.151	3,949	493.63
Estruch R, 2018, New Engl J Med	10.1056/NEJMoa1800389	2,795	349.38
Dinu M, 2018, Eur J Clin Nutr	10.1038/ejcn.2017.58	706	88.25
Cena H, 2020, Nutrients	10.3390/nu12020334	663	110.50
Martínez-González Ma, 2015, Prog Cardiovasc Dis	10.1016/j.pcad.2015.04.003	551	50.09
Guasch-Ferré M, 2021, J Intern Med	10.1111/joim.13333	535	107.00
Lane Mm, 2021, Obes Rev	10.1111/obr.13146	451	90.20
Godoy-Matos Af, 2020, Diabetol Metab Syndr	10.1186/s13098-020-00570-y	448	74.67
Cooper C, 2015, Am J Psychiat	10.1176/appi.ajp.2014.14070878	407	37.00
Anand Ss, 2015, J Am Coll Cardiol	10.1016/j.jacc.2015.07.050	359	32.64
Younossi Zm, 2021, Gastroenterology	10.1053/j.gastro.2020.11.051	356	71.20
Chen Xj, 2020, Nutr J	10.1186/s12937-020-00604-1	348	58.00
Masenga Sk, 2023, Int J Mol Sci	10.3390/ijms24097898	341	113.67
Van Horn L, 2016, Circulation	10.1161/CIR.0000000000000462	332	33.20
Amiot Mj, 2016, Obes Rev	10.1111/obr.12409	326	32.60
Esposito K, 2015, Bmj Open	10.1136/bmjopen-2015-008222	325	29.55
Rosato V, 2019, Eur J Nutr	10.1007/s00394-017-1582-0	318	45.43
Aune D, 2016, Bmc Med	10.1186/s12916-016-0730-3	316	31.60
Rees K, 2019, Cochrane Db Syst Rev	10.1002/14651858.CD009825.pub3	314	44.86
Castro-Barquero S, 2020, Nutrients	10.3390/nu12102983	305	50.83
Aleksandrova K, 2021, Redox Biol	10.1016/j.redox.2021.101869	303	60.60
De Toro-Martín J, 2017, Nutrients	10.3390/nu9080913	292	32.44
Salas-Salvadó J, 2019, Diabetes Care	10.2337/dc18-0836	286	40.86
Yang G, 2021, Metabolism	10.1016/j.metabol.2021.154712	280	56.00
Ndanuko Rn, 2016, Adv Nutr	10.3945/an.115.009753	268	26.80

**Figure 6:**
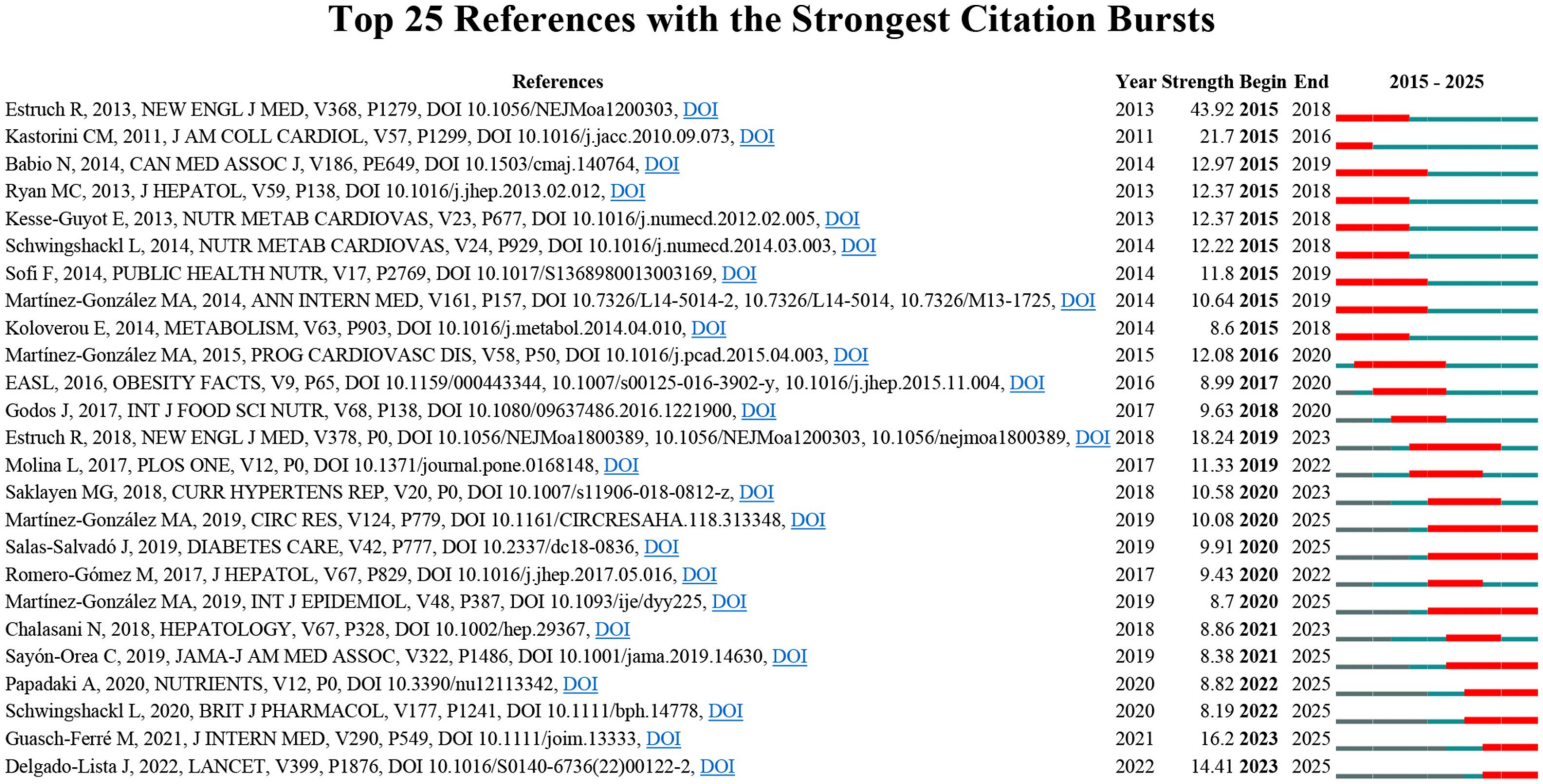
Top 25 references with the strongest citation bursts in MD in metabolic syndrome.

To accurately capture the research frontiers and hotspots in the MD and MetS field, CiteSpace software was used to identify the top 25 documents with the strongest citation bursts (Figure 6). The three documents with the highest burst strengths were: (1) “Primary Prevention of Cardiovascular Disease with a Mediterranean Diet” (burst strength: 43.92); (2) “The effect of Mediterranean diet on metabolic syndrome and its components: a meta - analysis of 50 studies and 534,906 individuals” (burst strength: 21.7); (3) “Primary Prevention of Cardiovascular Disease with a Mediterranean Diet Supplemented with Extra-Virgin Olive Oil or Nuts” (burst strength: 18.24). Additionally, the three most recently published documents among the citation burst list were: (1) “Long-term secondary prevention of cardiovascular disease with a Mediterranean diet and a low-fat diet (CORDIOPREV): a randomised controlled trial”; (2) “The Mediterranean diet and health: a comprehensive overview”; (3) “Mediterranean diet and health status: Active ingredients and pharmacological mechanisms.”

Combined analysis of highly cited documents and citation burst documents revealed that as global attention to metabolic health continues to grow, MD has gained widespread academic recognition as a potential dietary intervention. This study identified three core research directions in the field: 1. The effects of MD on metabolic indicators in MetS patients, including glycemic regulation, improvement of insulin resistance, correction of dyslipidemia, and management of hypertension. 2. The role of MD in MetS-related comorbidities, including the prevention and intervention effects on cardiovascular diseases, diabetes, and NAFLD. 3. The mechanisms underlying MD’s promotion of metabolic health, focusing on the anti - inflammatory and antioxidant effects of bioactive components such as polyphenols and unsaturated fatty acids, and the improvement of vascular endothelial function.

### 3.4 Keyword clusters and evolution of themes

**Figure 7:**
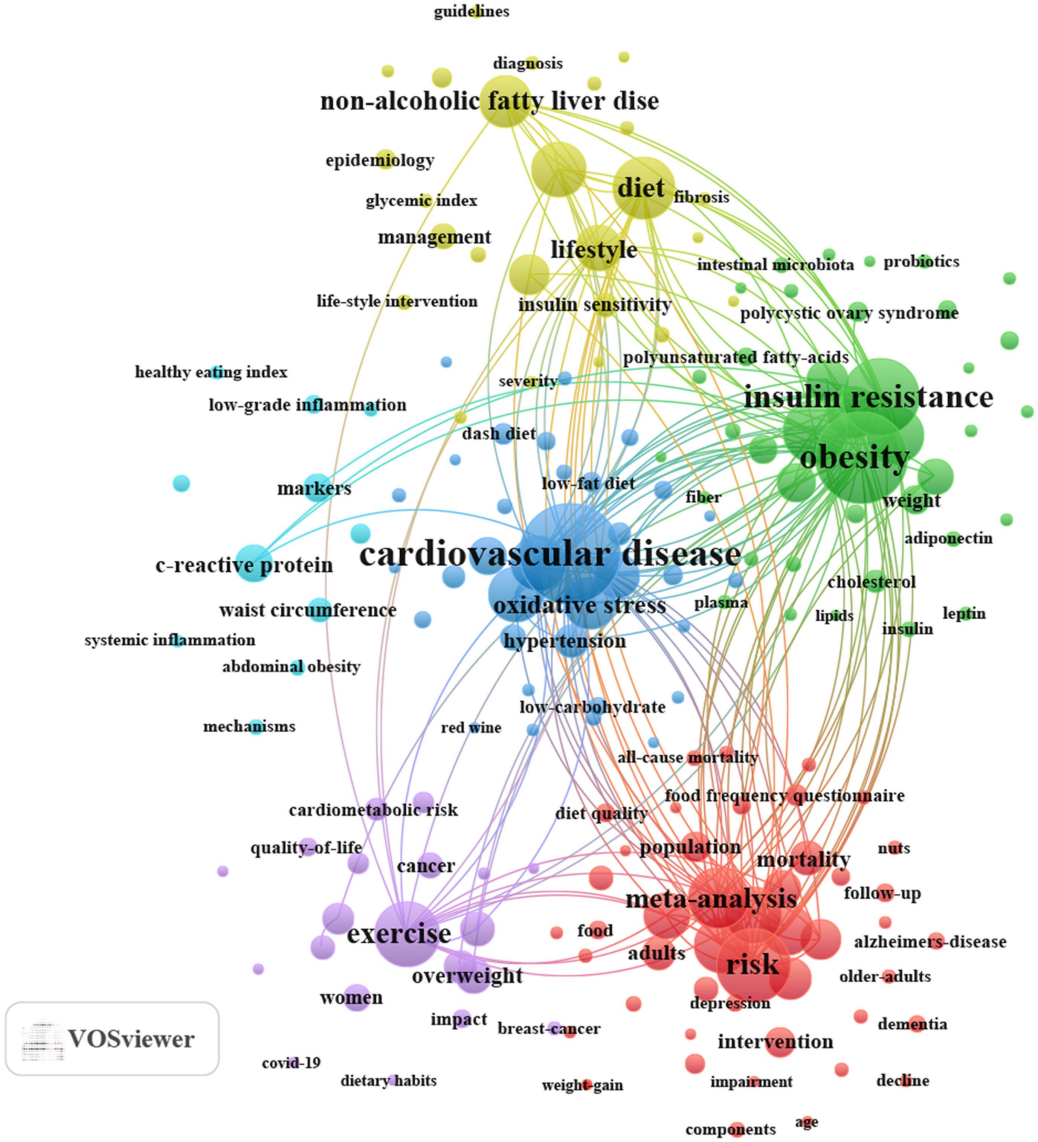
Keyword co-occurrence map of publications on MD in metabolic syndrome.

Keyword clusters are essential for rapidly grasping the main research themes and directions in a particular area. In our study, VOSviewer was used to identify 5,762 keywords. Table 6 presents the top 10 high-frequency keywords (frequency≥207). The most frequent keyword was “cardiovascular disease” (*n* = 509), followed by “obesity” (*n* = 439), “insulin resistance” (*n* = 335), “risk” (*n* = 320), “exercise” (*n* = 260), and “inflammation” (*n* = 245). Through cluster analysis, we observe six different colored clusters in Figure 7. (1) The impact of MD adherence on MetS-related cognitive impairment (red dots), including 46 keywords such as adherence, cognitive decline, Alzheimer’s disease, and components. (2) The effects of MD on MetS-related diabetes (green dots), including 39 keywords such as diabetes, insulin resistance, fatty acids, inflammation, and nutrients. (3) The impact of MD on MetS-related cardiovascular diseases and oxidative stress (blue dots), including 34 keywords such as cardiovascular disease, endothelial dysfunction, hypertension, antioxidant, and olive oil. (4) The effects of MD on MetS-related NAFLD and hepatic metabolism (yellow dots), including 25 keywords such as non-alcoholic fatty liver disease, steatohepatitis, vitamin-e, fibrosis, and insulin sensitivity. (5) The application and efficacy of MD in different MetS populations (purple dots), including 20 keywords such as men, dietary habits, adolescents, childhood obesity, and cancer. (6) The impact of MD on MetS-related central obesity and inflammation (light blue dots), including 11 keywords such as dietary inflammatory index, abdominal obesity, waist circumference, C-reactive protein, and systemic inflammation. All keywords included in the six clusters are detailed in Supplementary material 3. Notably, keywords related to inflammation, oxidative stress, insulin sensitivity, lipids, dietary quality, nutritional components, gut microbiota, and metabolism are distributed across different clusters, and the characteristics of interaction and overlap among these clusters indicate the core research hotspots in this field.

**Table 6:** Top 10 keywords related to MD in metabolic syndrome.

Rank	Keyword	Count
1	Cardiovascular disease	509
2	Obesity	439
3	Insulin resistance	335
4	Risk	320
5	Exercise	260
6	Inflammation	245
7	Diet	238
8	Meta-analysis	231
9	Adherence	208
10	Diabetes	207

To further expand the analytical dimensions, this study extracted 8,531 keywords from the Scopus database using VOS viewer software (Supplementary Figure S2). Current research hotspots in the field of MD and MetS mainly focus on two directions: (1) The regulatory effects of key nutritional components in MD (e.g., polyphenols, unsaturated fatty acids) on metabolic indicators, inflammatory responses, and cardiometabolic health; (2) The intervention effects and application value of MD on various metabolic diseases in different populations.

**Figure 8:**
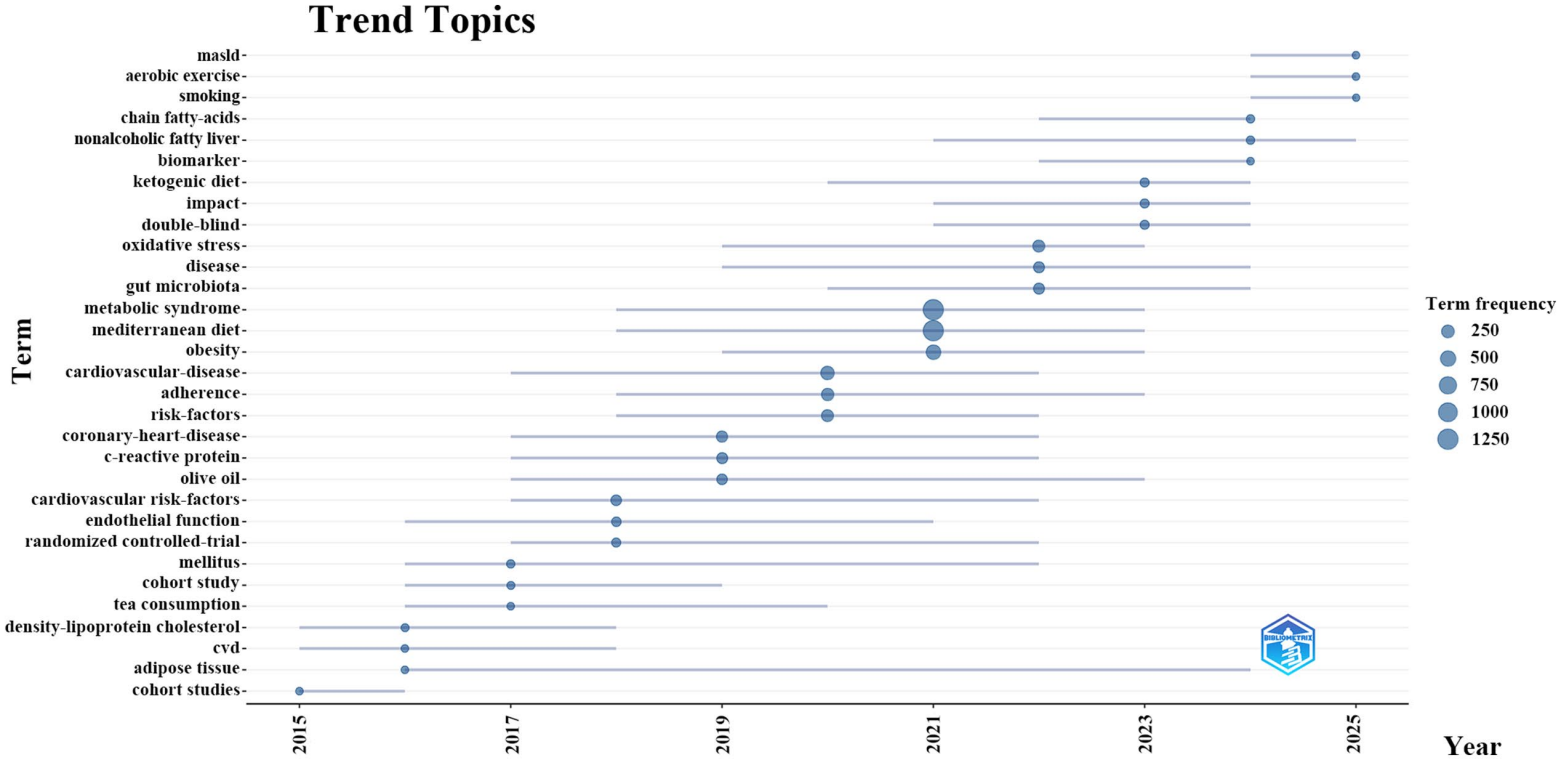
Trend topics in MD in metabolic syndrome.

To systematically predict future research trends in the MD and MetS field, the R language Bibliometrix package was used to generate a dynamic thematic evolution map (Figure 8). Such maps can effectively track the temporal development of research topics in specific fields and clearly present the evolutionary trajectory and inherent laws of research hotspots. Based on the map analysis, we summarized the phased evolutionary path of research directions in the field: From 2015 to 2017, the core research focus was on cohort-study design and traditional cardiometabolic indicators. Emphasis was placed on changes in indicators such as high-density lipoprotein cholesterol, cardiovascular disease risk factors, and C-reactive protein. The core exploration was on the potential mechanisms of MD in cardiovascular protection. From 2018 to 2020, the research focus shifted from correlation analysis to the explanation of intervention effects and pathological mechanisms. Themes such as randomized controlled trials, dietary adherence, metabolic syndrome, obesity, and oxidative stress appeared frequently, and emerging biological targets such as gut microbiota began to attract attention. From 2021 to 2023, research directions were further refined, focusing on specific dietary components (e.g., olive oil), NAFLD, and related biomarkers. Meanwhile, in-depth exploration was conducted on the regulatory effects of dietary structure and fatty acid types on cardiometabolic health. From 2024 to 2025, research showed a trend of interdisciplinary integration, with an increasing number of studies on combined interventions of lifestyle factors such as MASLD, aerobic exercise, smoking, and dietary patterns. This indicates that the field is moving toward multidimensional intervention and precise health management to more comprehensively address the complex pathological processes of MetS.

### 3.5 Comprehensive analysis of hotspots

In summary, this study systematically sorted out and clarified the core research hotspots and emerging development trends in the interdisciplinary field of MD and MetS through multidimensional comprehensive analyses, including identification of citation burst documents, keyword frequency statistics, keyword clustering analysis, and tracking of thematic dynamic evolution. Combined with the aforementioned bibliometric findings, the research hotspots in this field mainly focus on three core directions as follows: (1) Metabolic regulatory effects of MD on patients with MetS: Clarifying the specific roles and clinical application value of MD in glycemic control, insulin sensitivity, lipid metabolism, and blood pressure regulation. (2) Intervention effects of MD on MetS comorbidities and associated diseases: Focusing on the prevention, progression delay, and prognosis improvement of conditions including cardiovascular diseases, type 2 diabetes mellitus, NAFLD, and cognitive impairment. (3) Analysis of molecular regulatory mechanisms underlying MD-mediated metabolic health improvement: Further exploring the metabolic protective mechanisms of its key bioactive components (e.g., polyphenolic compounds, monounsaturated fatty acids, and omega-3 polyunsaturated fatty acids) through pathways such as anti-inflammation, anti-oxidative stress, improvement of vascular endothelial function, and regulation of gut microbiota homeostasis.

## 4 Discussion

### 4.1 General information

To systematically clarify the research hotspots and developmental trends of the MD in the field of MetS, this study used bibliometric analysis and data visualization methods to comprehensively collate 1723 relevant articles published in the Web of Science database between 2015 and 2025. Results showed that the number of publications in this field generally exhibited a significant upward trend, with a particularly prominent growth rate between 2016 and 2018, reflecting the continuous increase in academic attention to this interdisciplinary field. Notably, the number of relevant publications in 2022 and 2024 decreased, respectively, compared with the previous year. Two main factors account for this phenomenon: first, the impact of events occurring during a specific period—the global outbreak of COVID-19 in 2019 may have undermined the reliability of research findings, disrupted subsequent data collection and follow-up efforts, and thus impeded the smooth progress of relevant studies; second, the lag in database indexing, where some literature failed to be included in the statistical scope of the corresponding year due to delayed indexing.

In terms of international research contributions, Spain took a substantial lead with 383 papers, followed by Italy (299 papers) and the United States (47 papers). Among the top 10 institutions by publication output, 7 were from Spain, with 1 each from the United States, Greece, and Italy; notably, the Carlos III Health Institute of Spain ranked first with 175 publications. It is worth highlighting that 9 out of the top 10 institutions were located in Mediterranean coastal countries, with only 1 from the United States, indicating that Mediterranean coastal countries dominate research in this field. This phenomenon may be closely linked to geographical environments and dietary traditions: the local dietary patterns of Southern European Mediterranean coastal countries (e.g., Spain, Italy, and Greece) are highly consistent with the nutritional framework of MD, which not only facilitates the implementation of relevant studies but also improves participants’ dietary adherence more effectively.

In terms of journal distribution, the 1723 papers were published across 567 journals, with renowned journals including *Nutrients*, *Frontiers in Nutrition*, and *Antioxidants* contributing numerous high-quality research findings. Particularly noteworthy is that *Nutrients* has emerged as the core academic platform in this field; it has not only published a large volume of papers but also garnered a considerable number of citations. Its leading status not only highlights the journal’s academic influence in the field of MD and MetS but also confirms its vital role as a core dissemination vehicle for research outcomes in this domain.

### 4.2 Research hotspots and development trends

By analyzing the citation frequency, citation bursts, keyword occurrence rate, keyword clustering analysis, and keyword trends in the literature, we identified three main research focuses regarding the MD and MetS. The primary focus is the regulatory effect of MD on metabolic disorders. The second focus is the interventional role of MD in MetS comorbidities and related diseases. The third research hotspot is the mechanisms by which MD promotes metabolic health.

These findings are consistent with studies on MD promoting other health outcomes (Table 7). For example, a study published by Yan et al. (22) analyzed the research dynamics in the MD and diabetes mellitus, and found that components such as olive oil, legumes, and red wine are key research hotspots and trends; the core mechanisms mainly focus on anti-inflammatory and antioxidant effects, improvement of insulin sensitivity and secretion, regulation of lipid metabolism, and modulation of gut microbiota. In addition, according to the findings of Garrido-Romero et al. (23) the health-promoting potential of compounds such as polyphenols and phenolic acids, and the role of bioactive compounds in preventing and managing chronic diseases (including cardiovascular, metabolic, and neurodegenerative diseases) by regulating inflammation, oxidative stress, and gut microbiota are research hotspots in the field of olive-derived bioactive compounds. It is evident that key nutritional components, metabolic regulation, as well as mechanisms involving anti-inflammation, antioxidation, insulin sensitivity modulation, and gut microbiota modulation, represent a consensus of research hotspots in related fields. Meanwhile, this study emphasizes that in the field of the association between MD and MetS, the research focus is more centered on the metabolic benefits of MD for MetS and its intervention effects on related complications, and at the mechanistic level, it highlights the synergistic and precise regulatory mechanisms of multiple nutritional components.

**Table 7:** Core findings of similar bibliometric studies.

Study	Research topic	Research hotspots	Research focus
Present Study	MD and MetS	1. Metabolic regulatory effects of MD on MetS. 2. Synergistic regulation of polyphenols, unsaturated fatty acids, dietary fiber, and vitamins via anti-inflammatory, antioxidant, insulin sensitivity-improving, and gut microbiota-modulating pathways.	Focuses on the MetS field, emphasizing interventions against multiple related complications and the mechanisms of synergistic and precise regulation of diet-derived bioactive components.
Yan et al. (22)	MD and diabetes	1. Beneficial effects of olive oil, legumes, and red wine on diabetes. 2. Improvement of insulin sensitivity, anti-inflammatory, antioxidant, gut microbiota-modulating, and lipid metabolism-regulating effects.	Focuses on the diabetes field, emphasizing the metabolic benefits and regulatory mechanisms of specific foods.
Liu et al. (20)	MD and cancer	1. Mechanisms underlying the interaction between MD and gut microbiota and their impacts on various cancers. 2. Olive oil polyphenols as the primary bioactive components.	Focuses on the cancer field, emphasizing the effects of specific bioactive substances on cancer and the involved gut microbiota-related mechanisms.
Xu et al. (21)	MD and aging	1. Anti-aging mechanisms (inflammation, oxidative stress, and gut microbiome) and associations with age-related diseases (especially cardiovascular diseases, Alzheimer’s disease, and metabolic syndrome). 2. Interaction between the Mediterranean diet and gut microbiota.	Focuses on the aging field, emphasizing the effects of MD on aging and age-related diseases.
Gargi et al. (79)	Olive oil and cardiovascular disease	“Mediterranean diet,” “olive oil,” “cardiovascular disease,” “oxidative stress,” and “atherosclerosis” are key research topics.	Focuses on the cardiovascular disease field, emphasizing the effects and mechanisms of specific dietary patterns on cardiovascular diseases.
Garrido-Romero et al. (23)	Olive bioactive compounds	1. The health-promoting potential of compounds such as polyphenols, carotenoids, and secoiridoids. 2. Bioactive compounds prevent and manage chronic diseases, including cardiovascular, metabolic, and neurodegenerative diseases, mainly by regulating inflammation, oxidative stress, and the gut microbiota.	Focuses on olive-derived bioactive components, emphasizing the mechanisms by which polyphenols ameliorate cardiovascular, metabolic, and neurodegenerative diseases.

#### 4.2.1 Metabolic regulatory effects of MD in MetS patients

Comprehensive collation of bibliometric analysis results and existing clinical research evidence shows that the MD exerts significant positive regulatory effects on core metabolic dimensions of MetS patients, including blood glucose control, lipid metabolism optimization, insulin sensitivity enhancement, and blood pressure regulation.

The MD is characterized by low glycemic index (GI) foods, which helps reduce postprandial blood glucose fluctuations. Glycated hemoglobin (HbA1c) is a core indicator for assessing the average blood glucose level over the past 3 months. A study involving patients with prediabetes and type 2 diabetes showed that adhering to the MD can reduce HbA1c levels by an average of 7% compared with baseline (24). Moreover, the MD can regulate metabolic substrate preference, promoting the shift of body energy metabolism from glucose dependence to lipid oxidation, while activating peroxisome proliferator-activated receptor (PPAR)-related signaling pathways (25); this pathway can improve fat metabolism efficiency and promote visceral fat decomposition (26), and the reduction of visceral fat accumulation can break the vicious cycle of metabolic disorders and positively regulate glucose and lipid metabolism homeostasis (27). In terms of lipid optimization, the MD can reduce total cholesterol and triglyceride levels in MetS patients, moderately lower low-density lipoprotein cholesterol (LDL-C), and significantly increase high-density lipoprotein cholesterol (HDL-C), forming a lipid profile conducive to cardiometabolic health (28). Insulin resistance is a core pathological mechanism of MetS. The MD can enhance the sensitivity of peripheral tissues to insulin, reduce the increase in compensatory insulin secretion, and alleviate insulin resistance (29). In terms of blood pressure regulation, studies have shown that adhering to the MD for 3 months can reduce blood pressure levels in individuals with high-normal blood pressure or grade 1 hypertension, with a reduction in systolic blood pressure of up to 15.1 mmHg (30).

Although the metabolic regulatory benefits of the MD for MetS have been well established, personalized plans should be formulated based on patients’ metabolic characteristics (such as blood glucose fluctuation patterns and types of dyslipidemia) in clinical practice to avoid nutritional imbalances. On the other hand, core indicators including glycated hemoglobin (HbA1c), blood lipids, and liver and kidney function need to be regularly monitored during long-term implementation to promptly mitigate potential health risks (31). In addition, it is recommended that patients develop dietary plans under the collaborative guidance of medical experts and registered dietitians, and combine them with comprehensive measures such as exercise intervention to maximize the effectiveness of metabolic improvement while ensuring the safety and sustainability of the intervention (32).

#### 4.2.2 Effects of MD on common comorbidities and related diseases of MetS

MetS is often accompanied by multiple comorbidities, such as cardiovascular diseases, diabetes, NAFLD, and cognitive impairment. These diseases are interrelated and significantly increase health risks (33). MD has demonstrated clear clinical value in the prevention and intervention of the aforementioned comorbidities, and its mechanism of action is closely associated with metabolic regulatory effects.

Cardiovascular diseases, mainly coronary heart disease and cerebrovascular diseases, are the leading causes of death and disability worldwide, and MetS is a core risk factor for them (34). A multicenter randomized trial investigated the effect of MD on the primary prevention of cardiovascular disease. This trial enrolled 7,447 participants at high risk of cardiovascular disease but without cardiovascular disease (aged 55–80 years), with a median follow-up duration of 4.8 years. The study concluded that the Mediterranean diet was associated with a 30% reduction in the risk of major cardiovascular events (35). In secondary prevention, this dietary pattern can reduce the risk of adverse cardiovascular events such as myocardial infarction, ischemic stroke, and cardiovascular death in coronary heart disease patients by 24.7% ~ 28.1% (36), providing an important non-pharmacological intervention strategy for the rehabilitation management of clinical coronary heart disease patients. The MD can reduce the deposition of LDL-C in the vascular wall, inhibit vascular inflammation and atherosclerotic plaque formation (37, 38); at the same time, it can improve hemodynamic homeostasis, reduce target organ damage, repair impaired endothelium-dependent vasodilation function, and ultimately reduce the risk of progression of cardio-cerebrovascular diseases (39).

The occurrence of diabetes (especially type 2 diabetes) is closely related to dietary patterns, lifestyles, and metabolic abnormalities. The MD is an effective non-pharmacological intervention for the prevention and control of diabetes. Clinical studies have confirmed that the MD can exert significant metabolic protective effects, helping diabetic patients stabilize blood glucose fluctuations, improve insulin sensitivity, reduce HbA1c levels, and reduce dependence on hypoglycemic drugs (40); for healthy individuals or those with prediabetes, high compliance with the MD can reduce the risk of type 2 diabetes by 10% ~ 20% (41). In addition, this dietary pattern can maintain a healthy weight, reduce visceral fat accumulation, and delay the occurrence and development of diabetes and its complications (42). It should be noted that insufficient long-term compliance constitutes a major challenge in the application of the MD for diabetes management. Most patients find it difficult to adhere to this dietary pattern over the long term due to differences in eating habits and cultural backgrounds, which impairs its long-term intervention effects. Therefore, personalized plan adjustments and behavioral interventions are required to improve compliance.

NAFLD is characterized by excessive fat accumulation in hepatocytes, which can progress to cirrhosis or even liver cancer in severe cases. The MD has significant advantages in improving liver lipid metabolism. Studies have found that adherence to the MD is negatively correlated with serum alanine aminotransferase (ALT) levels, the degree of insulin resistance, liver fibrosis stage, and the severity of steatosis (43, 44), suggesting that it has hepatoprotective effects and can reduce liver damage. Relevant studies have shown that the MD can activate the expression of fatty acid oxidases mediated by PPARs, while downregulating the expression of sterol regulatory element-binding protein 1c (SREBP-1c) and lipid synthesis-related genes, thereby promoting liver fat decomposition (45, 46). In addition, this dietary pattern can reduce serum cholesterol, improve insulin sensitivity, and reduce the massive influx of free fatty acids into the liver, thereby alleviating liver fat accumulation (47).

Populations with multiple metabolic abnormalities experience faster cognitive decline, mainly manifested by decreased memory, attention, and executive function. The MD can exert cognitive protective effects through multiple pathways. Studies have shown that higher adherence to the MD is associated with a lower risk of cognitive decline and dementia (48). Neuroimaging evidence indicates that this dietary pattern is related to increased cortical thickness in the frontal, parietal, and occipital lobes of the brain, enlarged dentate gyrus volume, and enhanced brain network connectivity (49). Its potential mechanisms include promoting neural development and synaptic plasticity, improving vascular risk factors such as blood pressure and blood glucose, inhibiting neuroinflammation and oxidative stress, and reducing neuropathological protein load (50). However, there are still controversies regarding the conclusions of relevant studies. Research has suggested that the MD has no significant effect on delaying cognitive decline in healthy elderly populations (51). Its intervention effect may be affected by factors such as dietary servings, study design, intervention duration, ethnic differences, and geographical factors. The precise application value of the MD in the management of cognitive impairment still needs in-depth exploration.

Although MD has demonstrated significant clinical potential in the prevention and treatment of MetS-related comorbidities, large-scale, multi-ethnic, multi-center randomized controlled trials with participants from diverse cultural backgrounds are still required to verify the consistency of its efficacy across different global populations. Additionally, it is necessary to clarify the optimal dietary structure, intervention intensity, and duration for specific diseases. Meanwhile, long-term adherence remains a major bottleneck for clinical promotion. Research on a modified MD that adapts to local dietary cultures is needed to enhance feasibility and adherence while preserving its core nutritional characteristics. Moreover, it should further clarify the dose-effect relationship between dietary components and the prevention and control of comorbidities, thus providing a scientific basis for precise dietary intervention.

#### 4.2.3 Mechanistic research on MD promoting metabolic health

In the mechanism research on MD improving metabolic health, keyword clustering analysis showed that keywords related to nutrients, inflammation, oxidative stress, insulin sensitivity, lipids, diet quality, gut microbiota, and gut microbiota metabolites interact and overlap among different clusters, highlighting the multi-dimensional synergistic regulatory characteristics of the core mechanisms by which MD improves MetS. Specifically, inflammation, oxidative stress, and insulin resistance are core pathological links of MetS and related comorbidities. MD can simultaneously improve these pathological links, break the vicious cycle among them, and achieve systematic regulation of systemic metabolism. At the same time, the occurrence of nutrition-related keywords in different clusters confirms that beneficial components such as polyphenols, dietary fiber, unsaturated fatty acids, vitamins, and minerals rich in MD are the material basis for its multi-target intervention effects. Moreover, these nutritional components may further amplify anti-inflammatory and antioxidant effects through regulating gut microbiota composition and metabolite production, promoting metabolic health. In addition, the intersection of diet quality-related keywords with various clusters highlights that as an overall dietary pattern, the health benefits of MD not only depend on the role of a single nutritional component but also stem from the synergistic regulatory effects brought by optimized dietary structure, which provides a more comprehensive mechanistic support for the application of MD in non-pharmacological interventions for MetS.

Polyphenols are widely present in whole grains, vegetables, fresh fruits, and nuts in MD, and are one of the core components exerting metabolic protective effects. These substances exhibit significant antioxidant and anti-inflammatory effects, and help lower blood pressure, improve vascular function, and reduce the risk of atherosclerosis. In addition, polyphenols can improve insulin sensitivity, assist in regulating blood glucose levels, and have positive benefits for diabetic patients (52). Resveratrol can increase cellular glucose uptake by activating sirtuin 1 (SIRT1) and adenosine monophosphate-activated protein kinase (AMPK), thereby improving insulin sensitivity (53). Quercetin can inhibit the nuclear factor κB (NF-κB) inflammatory pathway, reduce the secretion of pro-inflammatory factors, and thus alleviate aging-related inflammation (54). Mammalian target of rapamycin (mTOR), as a core regulator of intracellular metabolism, can integrate multiple input signals such as nutritional status, energy levels, and stress signals to precisely regulate cell growth and metabolic levels (55, 56). Its dysfunction is closely related to metabolic disorders (57). One of the core mechanisms by which polyphenols improves metabolism is regulating the mTOR pathway, which improves insulin signal transduction efficiency, inhibits inflammation, improves liver glucose and lipid metabolism, and reduces the risk of MetS and related comorbidities (58). Polyphenols can also regulate gut microbiota structure, significantly increasing the abundance of probiotics such as Bifidobacterium (59). Improvements in gut microbiota bring multiple metabolic benefits, such as improving glucose tolerance, promoting insulin secretion, and reducing plasma cholesterol levels and systemic inflammatory responses (60).

MD is rich in fatty acids beneficial to metabolic health, including monounsaturated fatty acids (MUFA) and polyunsaturated fatty acids (PUFA). MUFA can improve metabolic disorders through the following mechanisms. First, it optimizes the lipid profile by regulating liver lipid synthesis and cholesterol transport, reducing blood LDL levels, and reducing the risk of atherosclerosis (61). Second, it promotes insulin signal transduction, reduces the release of toxic free fatty acids from adipose tissue, thereby enhancing insulin sensitivity and protecting pancreatic β-cell function (62). PUFA can be divided into n-3 polyunsaturated fatty acids (n-3 PUFA) and n-6 polyunsaturated fatty acids (n-6 PUFA). As anti-inflammatory lipids, n-3 PUFA can replace the production of pro-inflammatory mediators, and effectively alleviate metabolic inflammation by inhibiting the NF-κB pathway; it can also upregulate the expression of GLUT4 in skeletal muscle, reverse insulin resistance, and reduce the risk of type 2 diabetes (63, 64). The appropriate ratio of n-6 PUFA to n-3 PUFA synergistically improves metabolic disorders by regulating lipid metabolism, improving insulin signal transduction, and assisting in anti-inflammation (65). Studies have shown that MUFA and n-3 PUFA can selectively enrich beneficial intestinal bacteria, promote the production of short-chain fatty acids, improve intestinal barrier function, inhibit inflammation, and regulate blood glucose and lipids, thereby forming a “fatty acid-gut microbiota-metabolic homeostasis” regulatory axis (66, 67). Therefore, exploring the mechanism by which fatty acids mediate gut microbiota to improve MetS provides insights for future research development.

At the same time, the abundant dietary fiber (including soluble and insoluble dietary fiber) in MD also has multi-pathway synergistic regulatory characteristics, precisely targeting key pathological links of metabolic disorders. In terms of blood glucose regulation, soluble dietary fiber can form a viscous gel to delay the digestion and absorption of carbohydrates, reduce postprandial blood glucose fluctuations, and at the same time upregulate insulin receptor sensitivity, promote GLUT4 expression, and improve insulin resistance (68). In terms of lipid metabolism regulation, soluble dietary fiber can bind to bile acids and promote their excretion, reducing cholesterol reabsorption and lowering LDL-C levels (69); insoluble dietary fiber can increase chewing time and shorten colonic transit time, which can stimulate the vagus nerve to produce a sense of satiety, reduce calorie intake, and assist in improving obesity and insulin resistance (70). In addition, the fermentation of dietary fiber in the large intestine helps probiotics produce SCFAs, alleviate chronic inflammation, and promote systemic metabolic homeostasis (71). Therefore, the synergistic regulatory role of dietary fiber in maintaining metabolic homeostasis is a promising field for future research.

Vitamins A, C, D, and E in MD are important nutritional components. Among them, both vitamin C and vitamin E are efficient antioxidants, which can reduce oxidative stress-induced damage to pancreatic β-cells, reduce the production of lipid peroxides, promote insulin secretion and sensitivity, and at the same time inhibit the production of pro-inflammatory mediators, maintain vascular endothelial function, and assist in regulating lipid metabolism (72, 73). Vitamin A has the effects of inhibiting visceral fat accumulation, improving insulin receptor sensitivity, and enhancing glucose transport efficiency (74). Vitamin D regulates the expression of metabolism-related genes by binding to nuclear receptors, improves insulin resistance, and inhibits the NF-κB pathway to alleviate chronic inflammation (75). In addition, the low-sodium and high-potassium nutritional characteristics of the MD can reduce renal sodium reabsorption and prevent blood pressure elevation (76). Appropriate increase in calcium intake can reduce parathyroid hormone secretion and lower peripheral vascular resistance (77). Moreover, magnesium intake helps the synthesis of prostaglandin E, the relaxation of vascular smooth muscle, and blood pressure control (78).

However, through literature analysis, the mechanisms by which MD promotes metabolic health are not yet fully clear. The synergistic or interactive effects between polyphenols and between polyphenols and unsaturated fatty acids, as well as the mechanisms by which MD affects MetS through gut microbiota and insulin signal regulation, still need in-depth exploration. Therefore, in the future, multi-omics technologies should be integrated to accurately analyze the interactions between various dietary components and their overall effects on cellular signal networks, clarify the key targets of synergistic effects; in particular, gut microbiota and their metabolites, to deepen the understanding of related molecular pathways. In addition, it is necessary to explore the heterogeneous responses of different populations (such as different ethnic groups and different comorbidity types) to dietary components, providing a scientific basis for formulating precise nutrition plans.

### 4.3 Limitations

This study aims to enhance the understanding of the current development status and research hotspots in the field of MD promoting metabolic health, and explore potential research directions of great value. However, it should be noted that this study has several limitations. First, our research was restricted to reviews and articles published only in the English language, which may have led to the exclusion of certain high-quality papers. Second, our study included the WOS core nd Scopus database; other databases, such as PubMed, were not considered. As a result, the included literature production may be incomplete. Third, bibliometric analysis cannot evaluate methodological rigor, evidence level or conclusion credibility of single studies. Finally, our research only included articles published within a specific time period, which may lead to publication bias in the research results. Despite these limitations, this study still provides a comprehensive overview of the field, highlighting key issues and development trends. By comprehensively considering these factors, researchers can deeply understand the development context of the field, identify potential research directions, and use this information to guide future explorations.

## 5 Conclusion

Through bibliometric analysis, this study systematically reveals the key research hotspots and emerging frontier directions of MD in the field of MetS. The core knowledge system and research trends of this field are summarized as follows:

- The application of MD in dietary intervention for MetS has attracted widespread global attention. Countries such as Spain, Italy, the United States, Greece, and China are the core research forces in this field, with high research activity and close international cooperation.
- In this research field, Nutrients ranks first with the highest number of publications and citations, fully demonstrating its representative status as the core academic carrier in this field.
- Current research hotspots focus on the direct regulatory effects of MD on glycemic control, insulin sensitivity, lipid metabolism, and blood pressure in MetS patients.
- Currently, conducting MD intervention research around common comorbidities and related diseases of MetS, such as cardiovascular diseases, diabetes, NAFLD, and cognitive impairment, has become a significant trend in the field.
- Mechanistic research on MD promoting metabolic health mainly focuses on core nutritional components such as polyphenols, dietary fiber, unsaturated fatty acids, and vitamins, exploring their functional pathways such as anti - inflammation, anti - oxidation, and endothelial protection. Gut microbiota regulation has gradually become an emerging hotspot in mechanistic research.

In summary, this study provides systematic and key academic insights into the current research status, hotspot directions, and development trends of MD intervention in MetS. These findings help researchers quickly and accurately grasp the core dynamics of the field, clarify existing research gaps, and point out key directions for future research. At the same time, by revealing the limitations and potential innovation points of research in the field, this study provides targeted guidance for researchers to deepen their explorations, helping to promote the innovative development and breakthrough of research in this field.

## Data Availability

The original contributions presented in the study are included in the article/Supplementary material, further inquiries can be directed to the corresponding authors.

## Footnotes

1
http://www.bibliometrix.org
