# Supplementary material for: A bibliometric analysis of the Mediterranean diet in metabolic syndrome (2015–2025)
Source: Front Nutr. 2026 Jan 16;13:1765074. doi: 10.3389/fnut.2026.1765074 (PMC12855129; doi:10.3389/fnut.2026.1765074)
Supplement: Supplementary file 3 [file Image_1.pdf]

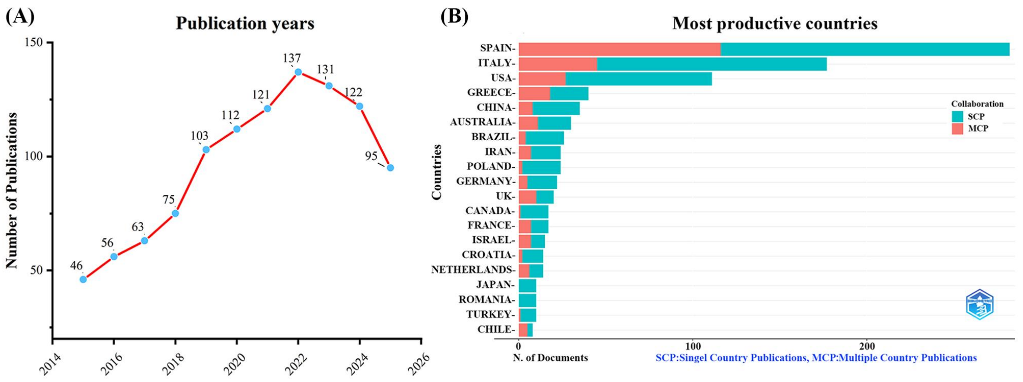

Figure S1 Trends in annual publication outputs on MD on metabolic syndrome from 2015 to 2025. (A) Trends of annual publication outputs. (B) Distribution of corresponding authors' countries and cooperation.

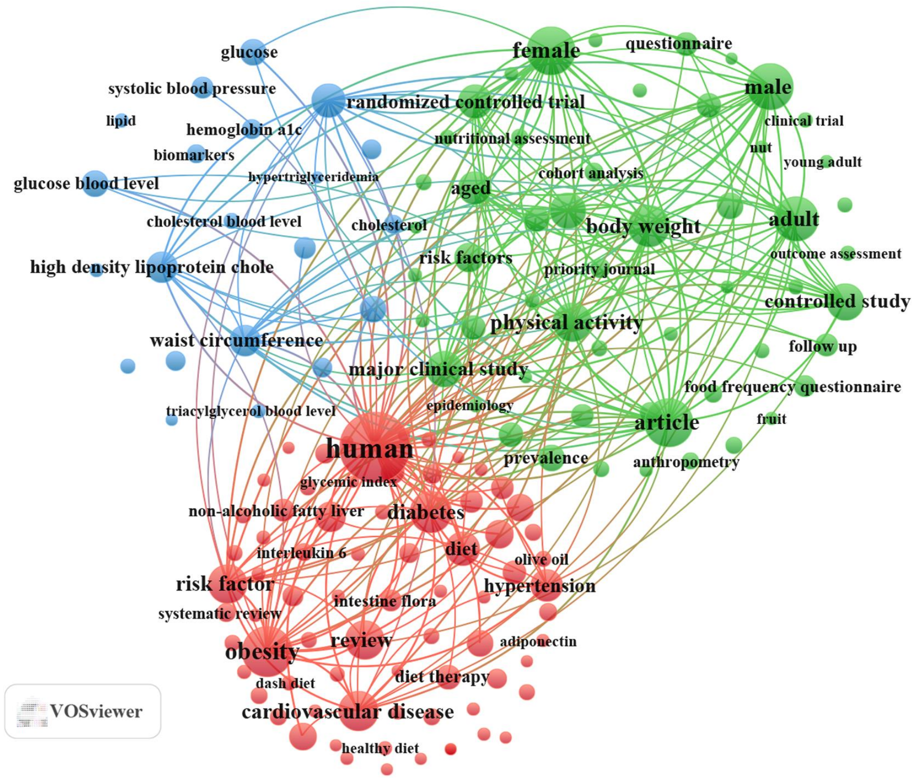

Figure S2. Keyword co-occurrence map of publications on MD on metabolic syndrome.
